# Maternal education inequalities in height growth rates in early childhood: 2004 Pelotas birth cohort study

**DOI:** 10.1111/j.1365-3016.2011.01251.x

**Published:** 2012-01-13

**Authors:** Alicia Matijasevich, Laura D Howe, Kate Tilling, Iná S Santos, Aluísio J D Barros, Debbie A Lawlor

**Affiliations:** a Postgraduate Programme in Epidemiology, Federal University of PelotasPelotas, Brazil; b School of Social and Community Medicine; c MRC Centre for Causal Analyses in Translational Epidemiology, University of BristolBristol, UK

**Keywords:** childhood height, Pelotas birth cohort, maternal education, child growth

## Abstract

Matijasevich A, Howe LD, Tilling K, Santos IS, Barros AJD, Lawlor DA. Maternal education inequalities in height growth rates in early childhood: 2004 Pelotas birth cohort study. *Paediatric and Perinatal Epidemiology* 2012; **26**: 236–249.

Socio-economic inequalities in attained height have been reported in many countries. The aim of this study was to explore the age at which maternal education inequalities in child height emerge among children from a middle-income country. Using data from the 2004 Pelotas cohort study from Brazil we modelled individual height growth trajectories in 2106 boys and 1947 girls from birth to 4 years using a linear spline mixed-effects model. We examined the associations of maternal education with birth length and trajectories of growth in length/height, and explored the effect of adjusting for a number of potential confounder or mediator factors.

We showed linear and positive associations of maternal education with birth length and length/height growth rates at 0–3 months and 12–29/32 months with very little association at 3–12 months, particularly in boys. By age 4 years the mean height of boys was 101.06 cm (SE = 0.28) in the lowest and 104.20 cm (SE = 0.15) in the highest education category (mean difference 3.14 cm, SE = 0.32, *P* < 0.001). Among girls the mean height was 100.02 cm (SE = 0.27) and 103.03 cm (SE = 0.15) in the lowest and highest education categories, respectively (mean difference 3.01 cm, SE = 0.31, *P* < 0.001). For both boys and girls there was on average a 3-cm difference between the extreme education categories. Adjusting for maternal height reduced the observed birth length differences across maternal education categories, but differences in postnatal growth rates persisted.

Our data demonstrate an increase in the absolute and relative inequality in height after birth; inequality increases from approximately 0.2 standard deviations of birth length to approximately 0.7 standard deviations of height at age 4, indicating that height inequality, which was already present at birth, widened through differential growth rates to age 2 years.

## Introduction

Childhood height is an important marker of health and living conditions in childhood, and secular trends in height have been shown to correlate with trends in economic development.^1^,^2^ Childhood height is strongly correlated with adult height, which in turn is associated with adult health and human capital.^3^ In a number of studies shorter stature has been found to be associated with lower intellectual performance, reduced work capacity, poor reproductive performance and increased risk of cardiovascular disease and type 2 diabetes.^4^^–^^7^ Positive attributes of height are more evident within than across populations. For example the Japanese are, by international standards, relatively short on average but have the longest life expectancy in the world.^8^ Height is influenced by a wide range of environmental factors experienced in childhood; these factors may be the determinants of the outcomes with which height is associated, rather than height *per se*. As such, height is considered not a causal factor that directly influences later health and other outcomes, but rather a marker of genetic and intergenerational/intrauterine factors and of childhood environmental exposures, and the timing of puberty.

Several prenatal and postnatal factors influence childhood height, including parents' height, genetics, and environmental factors such as parental social class and employment status, dietary intake and infectious diseases.^9^,^10^ Intergenerational influences on height have also been described, suggesting that a mother's intrauterine environment and her early development can directly influence her offspring's anthropometric outcomes.^11^ A study from the UK indicates that influences on a child's height may vary between different social and economic settings.^12^ Higher socio-economic position in general, and greater maternal education, in particular, has been shown to be robustly and strongly associated with better child health and survival.^13^ It is well known that socio-economic position influences childhood growth and attained adult height. Socio-economic inequalities in attained (adult) height have indeed been reported in many countries, but it has recently been suggested, based on observations in the UK, that socio-economic inequalities in adult height may narrow as countries undergo long-term economic development and consequently the majority of the population reach their genetic potential.^14^

The age at which socio-economic differentials in height appear and the patterns such differentials follow during childhood are less well known. Understanding whether these differentials are largely driven by intrauterine factors (resulting in birth length differences that persist or may be modified postnatally) or by postnatal factors (thus showing differentials in postnatal growth) is important for understanding how to reduce height differentials. Data from the Avon Longitudinal Study of Parents and Children (ALSPAC) from the UK showed that the socio-economic differential in height during childhood arises largely through inequalities in birth length, with negligible increases in the inequality from differences in growth after infancy.^15^ Conversely, Finch and Beck^16^ showed, among a nationally representative sample of 2- to 6-year-olds who were born in the US, strong socio-economic gradients in child height which remain consistent throughout early childhood. It is not known which pattern is found in lower-income settings, where childhood illness and malnutrition remain more common than in high-income countries and where, therefore, one might hypothesise that postnatal growth rates may be more strongly socio-economically patterned.

The aim of the present study was to investigate the age at which socio-economic inequalities in child height emerge among children from a middle-income country birth cohort study, the 2004 Pelotas cohort study from Brazil. The patterning of maternal education on individual growth trajectories was modelled, and potential factors that influence the maternal education–early childhood height growth association were examined.

## Methods

### Data source

During the whole of 2004, a population-based birth cohort study attempted to enrol all births from mothers resident in the urban area of the city of Pelotas, southern Brazil. Births were identified by daily visits to the five maternity hospitals. Mothers were interviewed soon after delivery. Information was obtained on demographic, environmental and socio-economic variables and on the characteristics of pregnancy, labour, delivery and health care service utilisation. In the city of Pelotas more than 99% of all deliveries take place in hospitals. In 2004, of the 4263 livebirths born to mothers living in the urban area of the city of Pelotas, 4231 were included in the perinatal study (0.8% loss) and were enrolled in the cohort study. Follow-ups were performed at home at mean (SD) ages 3.0 (0.1), 11.9 (0.2), 23.9 (0.4) and 49.5 (1.7) months. On each occasion, mothers were interviewed by trained fieldworkers and information about mother's and children's health was collected. A total of 3985, 3907, 3869 and 3799 children were visited at home at 3, 12, 24 and 48 months of age, respectively. Response rates were 95.7%, 94.3%, 93.5% and 92.0% for the 3-, 12-, 24- and 48-month follow-up, respectively. Further information about the methodology of the 2004 Pelotas birth cohort study is described in detail elsewhere.^17^

### Children's variables

Birth length was measured within 24 h of delivery by trained research fieldworkers following a standardised procedure using AHRTAG infantometers with 1-mm precision (AHRTAG baby length measures, London, UK).^18^ At each follow-up, anthropometric measurements were performed by trained fieldworkers with the children dressed in underwear and barefoot. Recumbent length (children ≤24 months of age) and standing height (48 months of age) were measured using a portable infantometer with 1-mm precision, custom-built for these studies.

Infant sex was recorded at birth. Estimates of gestational age were based on the last menstrual period (LMP), providing they were consistent with predicted birthweight, length and head circumference, based on the normal curves for these parameters for each week of gestational age. When LMP-based gestational age was unknown or inconsistent (*n* = 303 participants), we used the clinical maturity estimate based on the Dubowitz method,^19^ which was performed on almost all newborns.

Total breast-feeding duration (in months and days) was collected at each follow-up. The earliest available information on stopping breast feeding was used to reduce recall bias.

### Maternal variables

Information on maternal variables was gathered from the perinatal interview. Maternal schooling at the time of delivery was collected as a continuous variable and categorised according to the Brazilian Education System. The System is divided into three levels: fundamental (grades 1–8), intermediate (9–11) and higher education (≥12 years of formal education). Because of the small numbers of women without any formal education (0 years) and those with higher education, we opted to combine these women with the nearest category available. In addition, we decided to split the 1–8 category because it is very common in the city for women to start the fundamental level and only complete 4 years. Finally, maternal education was categorised as 0–4, 5–8 and ≥9 complete school years of formal education.

Family income in the month prior to delivery was expressed as multiples of the minimum wage per month (one minimum wage was worth approximately $80 in 2004). Maternal smoking behaviour during pregnancy was assessed retrospectively at birth and was self-reported. Regular smokers were those women who smoked at least one cigarette per day on an everyday basis in any trimester of pregnancy. Mother's skin colour was self-reported and categorised as white or black/mixed. Women who were single, widowed, divorced or lived without a partner were classified as single mothers. Maternal age in complete years was categorised as ≤19, 20–34 and ≥35 years. Parity was defined as the number of previous viable pregnancies and categorised as 0, 1 and ≥2. Maternal height was measured using a stadiometer manufactured in aluminium with 1-mm precision at the third-month follow-up. Information on paternal height was not available.

### Statistical analyses

We estimated individual growth trajectories using a linear spline mixed-effects model (two levels: measurement occasion and individual), fitted using the statistical package MLwiN version 2.20 (http://www.cmm.bristol.ac.uk/MLwiN/index.shtml). Such models allow for the change in scale and variance of height over time (i.e. they account for the fact that the mean height and the standard deviation of height increase as children get older) and use all available data from all eligible children under a missing-at-random assumption. They also allow for individual variation in growth trajectories, as random effects allow each individual to have different intercepts and slopes. Models for growth between birth and 48 months were constructed separately for boys and girls, for all individuals with data on maternal education and at least two measurements of length/height (*n* = 2106 boys and 1947 girls). The methodology identified spline points that defined periods of approximately linear growth based on the data. Based on previous work in the ALSPAC cohort,^15^ three spline points (four periods of linear growth) between birth and 48 months were chosen: 0–3 months, 3–12 months, 12–32 months, 32–48 months for girls, and 0–3 months, 3–12 months, 12–29 months and 29–48 months for boys. The children in the Pelotas cohort were measured, on average, at 0, 3, 12, 24 and 48 months. Thus the measurements at 3 and 12 months corresponded to the knot points. Although the fourth measurement was carried out on average at 24 months, we decided to retain the knot points in the model as 29/32 months in order to facilitate comparisons with ALSPAC and see how the inequalities in Brazil (a middle-income country) differ from those in the UK (a high-income country). This did not cause problems in the model, as although the mean age was 24 months, actual ages were scattered around this. Although derived in a contemporary UK cohort, these periods of linear growth have been demonstrated to be reasonably stable in different populations, including populations experiencing very diverse levels of deprivation and socio-economic conditions.^15^,^20^ Within this cohort we checked how well these spline points/growth periods fitted the observed data by comparing observed with predicted mean measurements in each period. Thus, five coefficients describe average growth in length/height in the cohort – birth length (i.e. the baseline measurement) and growth rates (cm/month) for the four periods described above.

Maternal education inequality in the growth trajectories was estimated by fitting interaction terms in the random-effects model between maternal education and the constant term (representing birth length) and each of the slopes for the four different growth periods. The parameters for these interaction terms demonstrate whether there are differences in birth length or growth in length/height in each period between groups of maternal education (i.e. the associated *P*-values test the null hypothesis of no difference in birth length or growth in each period by maternal education group).

We examined the effects of maternal education on birth length and growth in length/height accounting for potential confounders/mediators by using six different models: (1) adjusting for family income, (2) adjusting for marital status, maternal age, parity and maternal skin colour, (3) adjusting for maternal height, (4) adjusting for maternal smoking during pregnancy, (5) adjusting for gestational age and duration of breast feeding, and (6) adjusting for all significant confounders. To be included in the last model, variables had to be associated with maternal education and at least one of the outcomes (birth length and growth in length/height), with a *P* level <0.2. Variables included in the last model were family income, marital status, maternal skin colour and maternal height.

Several of the covariables considered in the current analyses could act through different pathways to confound or mediate the associations of maternal education with birth length and early childhood growth. Table S1 summarises our *a priori* conceptualisation of how these covariables might influence the associations we have examined. These are based on previous studies of the associations of each covariable with exposure (maternal education) and outcome (birth length and childhood growth). In the analytical model we considered maternal skin colour as a potential confounding factor of the maternal education–birth length and growth association. Maternal age at birth, maternal parity, smoking during pregnancy, gestational age and breast feeding were considered to be mediators. Family income, marital status and maternal height could possibly act as either confounder or mediator in this association.

### Ethics

The perinatal study and each follow-up were approved by the Research Ethics Committee of the Federal University of Pelotas School of Medicine. After being informed of the details of the study, mothers signed a form of informed consent for participation.

## Results

### Data and population

Data on growth and maternal education were available for 2106 boys and 1947 girls, representing 90% of the original 2004 Pelotas cohort. More than 86% of boys and girls had both data on birth length and measurements of length/height for all follow-ups (87.2% and 86.6% of boys and girls, respectively). Missing information for length/height was not associated with maternal education (*P* = 0.888), marital status (*P* = 0.155), maternal skin colour (*P* = 0.309), parity (*P* = 0.352), maternal smoking during pregnancy (*P* = 0.393), child's sex (*P* = 0.750), gestational age (*P* = 0.563) or duration of breast feeding (*P* = 0.652). However, missing information for length/height was more frequent among the richest group of women (*P* < 0.001) and among those ≥35 years old (*P* = 0.022).

Approximately 15% of children had mothers in the lowest education category and 44% in the highest category (Table 1). Most of the mothers lived with their partner (83.8%), were White (73.1%), primiparae (39.3%) and aged between 20 and 34 years old (67.8%). Children were born with a mean gestational age of 38 weeks and were breast fed on average for 10 months.

**Table 1:** Characteristics of participants included in study models

Variables	Participants *n* (%)
Maternal education (years) (*n* = 4053)	
0–4	621 (15.3)
5–8	1668 (41.2)
≥9	1764 (43.5)
Family income (minimum wage) (*n* = 4041)	
≤1.0	844 (20.9)
1.1–3.0	1872 (46.3)
3.1–6.0	919 (22.7)
6.1–10.0	223 (5.5)
>10.0	183 (4.5)
Marital status (*n* = 4053)	
Lived with partner	3398 (83.8)
Single mother	655 (16.2)
Maternal skin colour (*n* = 4053)	
White	2964 (73.1)
Black/mixed	1089 (26.9)
Maternal age (years) (*n* = 4051)	
≤19	771 (19.0)
20–34	2748 (67.8)
≥35	532 (13.1)
Parity (*n* = 4053)	
0	1592 (39.3)
1	1073 (26.5)
≥2	1388 (34.3)
Maternal smoking during pregnancy (*n* = 4053)	
No	2945 (72.7)
Yes	1108 (27.3)
Child's sex (*n* = 4053)	
Male	2106 (52.0)
Female	1947 (48.0)

	Mean (SD)
Maternal height (m) (*n* = 3974)	1.59 (0.06)
Gestational age (weeks) (*n* = 4045)	38.63 (2.26)
Duration of breast feeding (months) (*n* = 3772)	9.94 (8.90)

The association between potential confounder/mediator variables and birth length and growth in length in the different time periods is shown in Tables S2 and S3 for boys and girls, respectively.

### Model fit

Differences between observed and predicted measurements were very small in each period indicating good model fit (Table 2) and individual-level residuals were approximately normally distributed (Figures S1,S2).

**Table 2:** Comparing observed measurements with measurements predicted by the models

Growth period	No. measurements	Mean observed length/height cm (SD)	Mean difference (actual − predicted) cm [95% level of agreement]
Boys (*n* = 2106)			
Birth length	2159	48.55 (2.62)	−0.003 [−0.062 to 0.056]
0–3 months	1259	60.47 (2.66)	0.043 [−0.039 to 0.125]
3–12 months	2462	70.31 (7.25)	−0.040 [−0.101 to 0.022]
12–29 months	2371	85.46 (5.46)	0.012 [−0.036 to 0.059]
29+ months	1944	103.86 (4.75)	−0.003 [−0.023 to 0.016]
Girls (*n* = 1947)			
Birth length	2009	48.15 (2.41)	−0.001 [−0.006 to 0.004]
0–3 months	1173	59.05 (2.58)	0.001 [−0.007 to 0.009]
3–12 months	2213	68.95 (6.75)	−0.001 [−0.011 to 0.008]
12–32 months	2258	80.80 (6.09)	0.001 [−0.024 to 0.025]
32+ months	1806	100.24 (5.94)	−0.016 [−0.086 to 0.053]

SD, standard deviation.

### Maternal education differentials in birth length and growth trajectories

There was a positive gradient in birth length across categories of maternal education, with the lowest birth length among boys and girls from mothers in the lowest education category (*P*-values for interactions between birth length and maternal education 0.002 for boys and 0.040 for girls). There was a mean difference in birth length between the highest and lowest maternal education categories of 0.59 cm (0.23 standard deviations of birth length) and 0.43 cm (0.18 standard deviations of birth length) for boys and girls, respectively. These differences represent 1.2% and 0.9% of the average birth length of a son or a daughter, respectively, of a woman in the highest education category (Tables 3,4, Model 1).

**Table 3:** Mean (standard error) height growth rate across categories of maternal education among boys (*n* = 2106)

		Maternal education (years)				
Model	Age/growth period	0–4 Mean (SE)	5–8 Mean (SE)	9+ Mean (SE)	*P*-value^a^	Differences (9+) − (0–4)^b^
Model 1 (unadjusted)	Birth length (cm)	48.21 (0.15)	48.54 (0.08)	48.80 (0.08)	0.002	0.59
	Growth 0–3 months (cm/month)	3.87 (0.04)	4.01 (0.02)	4.13 (0.02)	<0.001	0.26
	Growth 3–12 months (cm/month)	1.61 (0.02)	1.61 (0.01)	1.63 (0.01)	0.147	0.02
	Growth 12–29 months (cm/month)	0.96 (0.01)	1.00 (0.01)	1.04 (0.01)	<0.001	0.08
	Growth 29–max months (cm/month)	0.56 (0.01)	0.55 (0.01)	0.56 (0.01)	0.606	0.00
Model 2 = Model 1 + family income	Birth length (cm)	48.08 (0.18)	48.40 (0.14)	48.58 (0.16)	0.028	0.50
	Growth 0–3 months (cm/month)	3.88 (0.05)	4.01 (0.04)	4.10 (0.04)	<0.001	0.22
	Growth 3–12 months (cm/month)	1.60 (0.02)	1.60 (0.02)	1.62 (0.02)	0.558	0.02
	Growth 12–29 months (cm/month)	0.95 (0.01)	0.98 (0.01)	1.01 (0.01)	<0.001	0.06
	Growth 29–max months (cm/month)	0.56 (0.01)	0.55 (0.01)	0.56 (0.01)	0.645	0.00
Model 3 = Model 1 + marital status, maternal age, parity, maternal skin colour	Birth length (cm)	48.20 (0.19)	48.61 (0.13)	48.81 (0.10)	0.004	0.61
	Growth 0–3 months (cm/month)	3.99 (0.05)	4.10 (0.03)	4.18 (0.03)	<0.001	0.19
	Growth 3–12 months (cm/month)	1.64 (0.02)	1.64 (0.01)	1.64 (0.01)	0.828	0.00
	Growth 12–29 months (cm/month)	0.99 (0.01)	1.03 (0.01)	1.03 (0.01)	<0.001	0.04
	Growth 29–max months (cm/month)	0.55 (0.01)	0.55 (0.01)	0.56 (0.01)	0.549	0.01
Model 4 = Model 1 + maternal smoking during pregnancy	Birth length (cm)	48.35 (0.16)	48.66 (0.10)	48.85 (0.08)	0.013	0.50
	Growth 0–3 months (cm/month)	3.89 (0.04)	4.02 (0.03)	4.13 (0.02)	<0.001	0.24
	Growth 3–12 months (cm/month)	1.61 (0.02)	1.61 (0.01)	1.63 (0.01)	0.229	0.02
	Growth 12–29 months (cm/month)	0.96 (0.01)	1.00 (0.01)	1.04 (0.01)	<0.001	0.08
	Growth 29–max months (cm/month)	0.56 (0.01)	0.55 (0.01)	0.56 (0.01)	0.611	0.00
Model 5 = Model 1 + maternal height	Birth length (cm)	48.42 (0.15)	48.59 (0.09)	48.69 (0.08)	0.290	0.27
	Growth 0–3 months (cm/month)	1.25 (0.38)	1.35 (0.38)	1.43 (0.39)	<0.001	0.18
	Growth 3–12 months (cm/month)	1.01 (0.16)	1.01 (0.16)	1.02 (0.16)	0.487	0.01
	Growth 12–29 months (cm/month)	0.31 (0.10)	0.34 (0.10)	0.38 (0.10)	<0.001	0.07
	Growth 29–max months (cm/month)	0.08 (0.07)	0.07 (0.07)	0.07 (0.07)	0.592	−0.01
Model 6 = Model 1 + gestational age and duration of breast feeding	Birth length (cm)	48.35 (0.13)	48.50 (0.08)	48.79 (0.07)	0.002	0.44
	Growth 0–3 months (cm/month)	4.22 (0.26)	4.36 (0.27)	4.48 (0.26)	<0.001	0.26
	Growth 3–12 months (cm/month)	2.94 (0.10)	3.00 (0.10)	2.98 (0.10)	0.076	0.04
	Growth 12–29 months (cm/month)	1.10 (0.07)	1.14 (0.07)	1.19 (0.07)	<0.001	0.09
	Growth 29–max months (cm/month)	0.60 (0.05)	0.60 (0.05)	0.61 (0.05)	0.591	0.01
Full model = Model 1 + family income, marital status, maternal skin colour and maternal height	Birth length (cm)	47.94 (0.21)	48.00 (0.18)	47.96 (0.20)	0.921	0.02
	Growth 0–3 months (cm/month)	3.91 (0.06)	4.04 (0.05)	4.12 (0.54)	<0.001	0.21
	Growth 3–12 months (cm/month)	1.59 (0.02)	1.59 (0.02)	1.60 (0.02)	0.619	0.01
	Growth 12–29 months (cm/month)	0.95 (0.02)	0.98 (0.01)	1.01 (0.01)	<0.001	0.06
	Growth 29–max months (cm/month)	0.57 (0.01)	0.57 (0.01)	0.58 (0.01)	0.387	0.01

a *P*-values relate to the comparison between maternal education categories, that is, they test the null hypothesis that there is no difference in growth rates between maternal education categories.

b Differences between the highest and lowest education levels.

**Table 4:** Mean (standard error) height growth rate across categories of maternal education among girls (*n* = 1947)

		Maternal education (years)				
Model	Age/growth period	0–4 Mean (SE)	5–8 Mean (SE)	9+ Mean (SE)	*P*-value^a^	Differences (9+) − (0–4)^b^
Model 1 (unadjusted)	Birth length (cm)	47.54 (0.15)	47.81 (0.09)	47.97 (0.09)	0.040	0.43
	Growth 0–3 months (cm/month)	3.69 (0.04)	3.77 (0.02)	3.93 (0.02)	<0.001	0.24
	Growth 3–12 months (cm/month)	1.55 (0.02)	1.58 (0.01)	1.61 (0.01)	0.002	0.06
	Growth 12–32 months (cm/month)	0.99 (0.01)	1.03 (0.01)	1.06 (0.01)	<0.001	0.07
	Growth 32–max months (cm/month)	0.47 (0.01)	0.47 (0.01)	0.47 (0.01)	0.884	0.00
Model 2 = Model 1 + family income	Birth length (cm)	47.29 (0.18)	47.47 (0.14)	47.50 (0.16)	0.505	0.21
	Growth 0–3 months (cm/month)	3.70 (0.05)	3.78 (0.04)	3.94 (0.04)	<0.001	0.24
	Growth 3–12 months (cm/month)	1.55 (0.02)	1.57 (0.02)	1.59 (0.02)	0.086	0.04
	Growth 12–32 months (cm/month)	0.99 (0.01)	1.02 (0.01)	1.04 (0.01)	<0.001	0.05
	Growth 32–max months (cm/month)	0.48 (0.01)	0.48 (0.01)	0.48 (0.01)	0.730	0.00
Model 3 = Model 1 + marital status, maternal age, parity, maternal skin colour	Birth length (cm)	47.49 (0.18)	47.85 (0.11)	48.00 (0.1)	0.030	0.51
	Growth 0–3 months (cm/month)	3.81 (0.05)	3.85 (0.03)	4.00 (0.03)	<0.001	0.19
	Growth 3–12 months (cm/month)	1.61 (0.02)	1.62 (0.01)	1.63 (0.01)	0.490	0.02
	Growth 12–32 months (cm/month)	1.03 (0.01)	1.06 (0.01)	1.08 (0.01)	0.001	0.05
	Growth 32–max months (cm/month)	0.46 (0.01)	0.46 (0.01)	0.47 (0.01)	0.626	0.01
Model 4 = Model 1 + maternal smoking during pregnancy	Birth length (cm)	47.77 (0.16)	48.02 (0.10)	48.07 (0.09)	0.251	0.3
	Growth 0–3 months (cm/month)	3.71 (0.04)	3.79 (0.03)	3.94 (0.02)	<0.001	0.23
	Growth 3–12 months (cm/month)	1.55 (0.02)	1.58 (0.01)	1.61 (0.01)	0.001	0.06
	Growth 12–32 months (cm/month)	1.01 (0.01)	1.04 (0.01)	1.07 (0.01)	<0.001	0.06
	Growth 32–max months (cm/month)	0.48 (0.01)	0.47 (0.01)	0.47 (0.01)	0.898	−0.01
Model 5 = Model 1 + maternal height	Birth length (cm)	47.83 (0.15)	47.85 (0.09)	47.85 (0.08)	0.993	0.02
	Growth 0–3 months (cm/month)	2.74 (0.36)	2.81 (0.36)	2.97 (0.37)	<0.001	0.23
	Growth 3–12 months (cm/month)	0.64 (0.15)	0.65 (0.15)	0.67 (0.15)	0.140	0.03
	Growth 12–32 months (cm/month)	0.41 (0.09)	0.43 (0.10)	0.46 (0.10)	<0.001	0.05
	Growth 32–max months (cm/month)	0.20 (0.09)	0.19 (0.09)	0.19 (0.09)	0.631	−0.01
Model 6 = Model 1 + gestational age and duration of breast feeding	Birth length (cm)	47.66 (0.14)	47.80 (0.08)	47.94 (0.08)	0.136	0.28
	Growth 0–3 months (cm/month)	4.57 (0.24)	4.66 (0.24)	4.83 (0.24)	<0.001	0.26
	Growth 3–12 months (cm/month)	2.41 (0.10)	2.44 (0.10)	2.48 (0.10)	<0.001	0.07
	Growth 12–32 months (cm/month)	1.05 (0.06)	1.09 (0.06)	1.12 (0.06)	<0.001	0.07
	Growth 32–max months (cm/month)	0.51 (0.06)	0.51 (0.06)	0.51 (0.06)	0.879	0.00
Full model = Model 1 + family income, marital status, maternal skin colour and maternal height	Birth length (cm)	47.48 (0.17)	47.62 (0.15)	47.72 (0.19)	0.340	0.24
	Growth 0–3 months (cm/month)	3.71 (0.05)	3.79 (0.04)	3.94 (0.04)	<0.001	0.23
	Growth 3–12 months (cm/month)	1.55 (0.02)	1.57 (0.02)	1.59 (0.02)	0.088	0.04
	Growth 12–32 months (cm/month)	1.00 (0.01)	1.03 (0.01)	1.05 (0.01)	0.001	0.05
	Growth 32–max months (cm/month)	0.47 (0.01)	0.47 (0.01)	0.47 (0.01)	0.527	0.00

a *P*-values relate to the comparison between maternal education categories, that is, they test the null hypothesis that there is no difference in growth rates between maternal education categories.

b Differences between the highest and lowest education levels.

There is evidence of different growth velocity according to categories of maternal education for some, but not all, growth periods. Among boys in the first 3 months of life and between 12 and 29 months, growth rates tended to be higher in the highest maternal education category. Among girls, the growth rate in the first 32 months of life was higher in the highest maternal education category (Tables 3,4, Model 1).

Among boys, adjusting growth rate for family income (Table 3, Model 2), marital status, maternal age, parity and maternal skin colour (Table 3, Model 3) and maternal smoking during pregnancy (Table 3, Model 4) did not substantially change differences in either birth length or growth rates across categories of maternal education. When maternal height was adjusted for (Table 3, Model 5), the birth length differences across maternal education categories were reduced, but maternal education differences in growth rates persisted and were of similar relative magnitude to the unadjusted analysis (Table 3, Model 1). Adjusting for the child's characteristics (gestational age and duration of breast feeding) did not affect growth rate trends across maternal education categories (Table 3, Model 6). Adjusting for all potential confounders (family income, marital status, maternal skin colour and maternal height) reduced maternal education differences in birth length without affecting maternal education differences in growth rates in the first 3 months of life and between 12 and 29 months (Table 3, Full model).

Among girls, differences in birth length across maternal education categories disappeared after adjusting for family income (Table 4, Model 1). Adjusting growth for marital status, maternal age, parity and maternal skin colour (Table 4, Model 2) and maternal smoking during pregnancy (Table 4, Model 3) did not substantially change differences across categories of maternal education in either birth length or growth rates. When maternal height was adjusted for (Table 4, Model 5), birth length differences across maternal education categories disappeared and growth rates in the 0–3 and 12–32 months periods were reduced, although to a lesser extent than was observed among the boys. Adjusting for gestational age and duration of breast feeding did not alter the pattern of birth length or growth rates across categories of maternal education (Table 4, Model 6). Adjusting for all potential confounders reduced maternal education differences in birth length without affecting maternal education differences in growth rate trends in the 0–3 and 12–32 months periods (Table 4, Full model).

**Figure 1:**
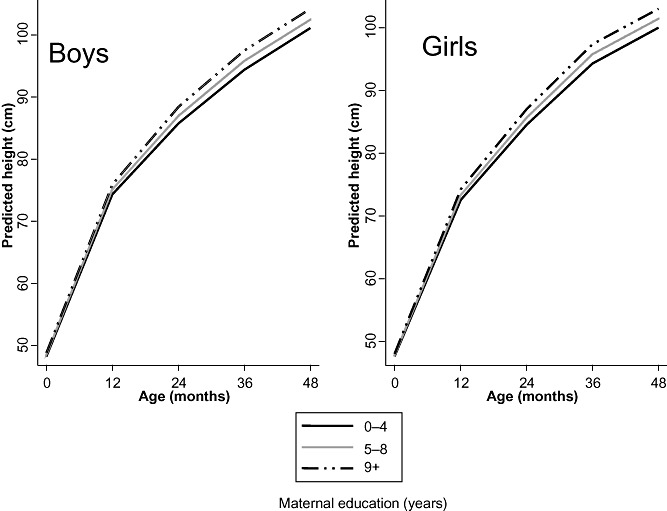
Average height (cm) trajectories of boys and girls predicted by the multilevel models according to maternal education categories (0–4, 5–8 and 9+ years of formal education).

Final predicted heights displayed in Table S4 were derived from the results of the multilevel linear spline model and were used to build Figure 1. That figure shows the average predicted growth trajectories for boys and girls according to each category of maternal education. By age 4, the mean predicted height of boys in the lowest education category was 101.06 cm (SE = 0.28) compared with 104.20 cm (SE = 0.15) in the highest education category (mean difference 3.14 cm, SE = 0.32, *P* < 0.001, representing 0.65 standard deviations of height at age 4) (Table S4). The equivalent predicted heights at age 4 for girls were 100.02 cm (SE = 0.27) and 103.03 cm (SE = 0.15) in the lowest and highest education categories respectively (mean difference 3.01 cm, SE = 0.31, *P* < 0.001, representing 0.68 standard deviations of height at age 4). Thus for both boys and girls there was, on average, a 3-cm difference between the extreme maternal education categories, representing 2.9% of the average height of a son or a daughter of a woman in the highest education category.

## Discussion

In a middle-income country, we have shown linear and positive associations of maternal education with birth length and growth rates in length/height at 0–3 months and 12–29/32 months with very little association between 3 and 12 months, particularly in boys.

Our data demonstrate an increase in the absolute and relative inequality in height after birth; inequality increases from approximately 0.2 standard deviations of birth length to approximately 0.7 standard deviations of height at age 4. This indicates that height inequality, which was already present at birth, widened as a result of differences in growth rates to age 2 years. Even though adjusting for maternal height reduced the observed birth length differences across maternal education categories, differences in postnatal growth rates among some, but not all, growth periods persisted. Other factors considered as potential confounders or mediators of the maternal education–growth associations (family income, marital status, maternal age, parity, maternal skin colour, maternal smoking during pregnancy, gestational age, breast-feeding duration) explained little of the observed associations with growth, although family income did reduce maternal education differences in birth length in girls.

A major strength of the present study was the mode of data collection; prospective information was obtained among a large unselected population combined with the use of standardised anthropometric measurements performed by trained fieldworkers, with high follow-up rates and low levels of missing data for most variables. However, some methodological difficulties of the study need to be discussed. First, children for whom we have complete information on growth were of lower-income and younger mothers compared with those with missing information for anthropometric variables. It is possible that the magnitude of maternal education inequalities in birth length and growth in length/height could have been higher if all cohort members had been considered. Second, measures for early childhood illness and malnutrition were not included in the present analyses and their effect on maternal education inequalities in height could not be explored. We believe that this is an area for further work. Third, we did not include time-varying covariates in the analyses, although it is possible that baseline levels of maternal variables (i.e. family income, maternal education, marital status) could have changed throughout the 4-year follow-up. However, we wished to avoid the possibility of reverse causality of child health influencing parental socio-economic position, so we chose maternal education and family income variables that were measured at the birth of the child. Finally, many other methods are available for modelling growth. Conditional growth modelling has been used to investigate patterns of child growth in length/height in early childhood and final attained stature in the COHORTS (Consortium on Health-Orientated Research in Transitional Societies) collaboration.^21^ Latent class analysis was used to examine the association of growth trajectory from birth to 12 months with subsequent hospital admissions in the ‘Children of 1997’ Chinese birth cohort.^22^ In the present study we have assumed a biologically implausible piecewise linear relationship between height growth and age. More plausible curvilinear forms (such as non-linear spline,^23^ complex polynomial or other non-linear models^24^,^25^) would have resulted in closer approximation to the growth measures. The association between maternal education and these more complex models, however, would have been far less easy to interpret. Our model allowed us to derive very simple and easily interpretable associations between maternal education and the rate of growth in given periods of early childhood. Our approach simplified the length/height growth trajectories by using a linear spline model while retaining good fit between observed and predicted values. We believe that the methods presented here are a useful compromise between perfect modelling of growth, and interpretable summaries of growth which can be related to exposures. The knot points used in the linear spline models for these analyses were knot points that had been derived in the ALSPAC cohort study, which recruited pregnant women in the south-west of England in the early 1990s. In ALSPAC considerably more data points were available (median number of seven measurements per child) and these were collected at varying ages for each child. The knot points were derived using fractional polynomials in such a way that growth was approximately linear between these points; the details of the statistical methodology are published elsewhere.^15^ Because we had fewer data collection points in the Pelotas cohort, and these were evenly spread, we were unable to derive Pelotas-specific knot points for this cohort and hence used those that had already been derived in ALSPAC. Although the ALSPAC cohort is in a different setting to the Pelotas cohort, we are confident that the knot points in the linear spline model are appropriate to the Brazilian cohort study for several reasons. First, the fit of the Pelotas data to the model is very good. Second, knot points at very similar ages have also been identified using similar methods to those used in ALSPAC in two additional cohorts from very different settings: (a) the Barry Caerphilly study cohort of children from a deprived area of South Wales in the 1970s,^20^ and (b) the Promotion of Breastfeeding Intervention Trial, a cohort of children born in Belarus in 1996/1997.^26^ Finally, the WHO Child Growth Standards study demonstrated that children tend to follow similar growth patterns across diverse settings.^27^

Socio-economic inequalities in height among children have been previously described among high-income^12^,^15^,^28^,^29^ as well as low- and middle-income countries.^30^^–^^34^ However, this association has not been confirmed everywhere. Rona *et al*.^35^ showed that socio-economic inequalities in child height in Trinidad and Tobago were of marginal importance; among all socio-economic variables analysed (parental education, employment status and ethnic background), only piped water supply was associated with children's height. Data from two longitudinal birth cohorts, the Birth to Twenty study in South Africa and the Cebu Longitudinal Health and Nutrition Survey in the Philippines, showed that the association between proxy measures of household socio-economic status and stunting in childhood was context-specific.^36^ Most of the studies investigated patterns of height inequality using cross-sectional data and to our knowledge few studies, and none in a low- or middle-income country setting, have examined inequalities with childhood growth in height using longitudinal data. A study with data from Mozambique showed that on average, one additional year of maternal schooling was associated with the child's height-for-age *z*-score higher by nearly 0.031 and that a child whose mother has completed 7 years of primary schooling has a height-for-age *z*-score 0.22 higher than a child whose mother has never attended school.^30^ Patel *et al*.^37^ using data from 6.5-year-olds from the Republic of Belarus showed that the difference in standing height, leg length and trunk length between children of mothers with initial/incomplete/common secondary and those of mothers that completed university was 1.86 cm [95% confidence interval (CI) 1.46, 2.25], 0.91 cm [95% CI 0.67, 1.14] and 0.95 [95% CI 0.71, 1.19], respectively. A recent study of 12 366 children from the ALSPAC cohort analysed height trajectories from birth to 10 years using the same methodology as in the present study.^15^ In agreement with those investigators, our study found a positive gradient in birth length across categories of maternal education for boys and girls. Even though the mean birth length difference between babies born to mothers in the highest and lowest categories of maternal education was almost the same in both cohort studies (0.53 and 0.51 cm for Pelotas and ALSPAC, respectively), the differences in growth rates across maternal education categories were higher in the Pelotas cohort study. In a nationally representative sample of children from the US (mean age 3.66, SD 1.33), investigators showed that each additional year of maternal education was associated with a mean height-for-age *z*-score higher by 0.024 standard deviations; in other words, a child of a mother with a college degree was, on average, 1.02 cm taller than a child whose mother had only a high school degree.^16^

Our study showed, by the age of 4 years, higher inequalities in height between children in the lowest and highest maternal education categories than in the other two studies previously mentioned (mean height difference of 3 cm in Pelotas and 1 cm in the US and ALSPAC study). Although in the ALSPAC study most of the educational inequality in height during childhood was driven by differences in birth length, this pattern was observed neither in the US study, nor in the Pelotas cohort, where height inequality remained or widened during early childhood.

Maternal education in the Pelotas study was measured as complete years of schooling, while in the ALSPAC study it was measured as educational achievement, with all mothers in the ALSPAC cohort having at least 12 years of compulsory schooling (more than the highest category of years of schooling used in our analyses for the Pelotas cohort). Schooling and educational achievement do not mean exactly the same thing,^38^ and thus the measures between the two cohorts are not directly comparable. It is possible that stronger education-related inequalities in height would exist in the Pelotas cohort study between children of women who do and do not have higher qualifications. It appears that inequalities in attained height within the ALSPAC cohort are likely to be largely driven by intergenerational/intrauterine factors, whereas in the Pelotas cohort and in the study from the US postnatal factors also make an important contribution to maternal education differentials in attained height as a consequence of their influence on postnatal growth.

Many studies have demonstrated a strong correlation between maternal education and child health. However, some investigators disagree with the idea of a causal effect of maternal education on child health and survival.^39^ But how could maternal education improve child height? Thomas *et al*.^40^ using data from the 1986 Brazilian Demographic and Health Survey demonstrated that mother's education had a large and significant association with child height in both the rural and urban sectors of the north-east region of Brazil. Almost all the effect of maternal education was explained by higher access to information and, even though it was not possible to discern the exact type of information which was important, they concluded that the availability and processing of information played a critical role in the transmission of the benefits of education. Maternal education benefits could operate through adopting favourable behaviours or embracing the use of modern health services (as a result of accessing and using information), which could improve child health and hence contribute to growth.

Our results showed that the association of maternal education with birth length and growth rates during early childhood does not appear to operate through income as birth length and growth rate differentials across maternal education categories remained almost the same after adjusting for family income, with the exception of the association with birth length in girls. The relationship between family income and disposable material resources could be influenced by mean family size and sources of unofficial income, which were not accounted for in this study. Other measures which are commonly used to describe socio-economic status, like parental employment and ownership of domestic appliances that were not used in the present study, could have shown different associations with birth length and growth.

Leg length is a key component of early height. While in long-term industrialised populations it has been shown that leg length is particularly sensitive to postnatal environmental influences,^41^ these results have not been confirmed in other settings. A recent study reporting results of a community trial of nutritional supplementation given to pregnant women and young children in India showed that the relative trunk length, and not the leg length, was the component of height most associated with the intervention.^42^ Among Chinese women aged at least 50 years, childhood socio-economic conditions were not associated with leg length, indicating that leg length is not a universal biomarker of early-life conditions.^43^

In our study maternal education was consistently associated with differences in growth rates at age 0–3 months and 12–29/32 months, but not at all after 29/32 months. A possible explanation could be that paternal influences are stronger at older ages. One study from France suggested that mother's height influenced child's height velocity in the first months, but paternal height was more important in the second year of life.^44^ According to the Karlberg infancy–childhood–puberty growth model, there are at least three endocrine phases of linear growth from birth to maturity: infancy (which includes fetal growth), childhood and puberty, with key hormones or growth-promoting systems involved in each component.^45^

The maternal contribution to her offspring's length reflects both genetic and intergenerational/intrauterine factors (see Table S1). Maternal height represents a highly complex combination of genes and environment in the mother's own intrauterine period and childhood. Maternal height could be linked to childhood length via genetic variants; however, it could also represent a perpetuation of a programming influence through several generations.^46^ In addition, epigenetic influences could constrain pre-pubertal growth as has been reported in a recently reported Chinese population.^47^ In our study birth length differentials across categories of maternal education disappeared after adjustment for maternal height, indicating that inequalities in birth length were explained by inequalities in maternal height. However, differentials in growth rates across maternal education categories in the 0–3 months period remained practically unchanged after adjusting for maternal height, indicating that other factors – presumably postnatal environmental ones – may have an import role in this period of time.

Even though shorter people, like the Japanese, have a greater longevity potential than many other taller populations, within populations child height is considered to be a strong predictor of human capital and the health of future generations. Evidence to date suggests that socio-economic inequalities in childhood height have been reduced in magnitude in some settings in the last decades. Li *et al*.,^29^ using information from the 1958 British cohort members and their offspring, showed that inequalities in height narrowed over time in Great Britain. Monteiro *et al*.,^33^ using data from four nationwide probability household surveys covering a 33-year period in Brazil, documented major reductions in socio-economic inequalities in stunting between poor and wealthy children. However, in spite of these improvements, our study shows that socio-economic inequalities in childhood height and growth rates still exist and are of significant magnitude.

Even though we cannot deduce exactly the reason why higher formal education would make such a difference in child height, our findings accentuate the importance of reducing socio-economic inequalities in child outcomes in low- and middle-income populations like Brazil. Improving educational standards may be one way of reducing inequalities. Much progress has been made in Brazil in the last decades to ensure universal access to primary education and to improve the quality of schools around the country. However, data from the 2004 Pelotas cohort study indicated that almost one in 10 mothers did not complete primary education. The second Millennium Development Goal states that boys and girls everywhere will be able to complete a full course of primary schooling. This measure should help to reduce inequalities in childhood height, among other aspects of child health, and thence to avoid possible long-term consequences of impaired linear growth to adult life in future generations.

## Author's contribution

A. M. identified the research question, conducted the analyses and wrote the first draft of the article. LDH contributed with the analyses, the interpretation of the findings and the writing of the article. KT, DAL, ISS and AJDB contributed to developing the research question, the interpretation of the analysis and assisted with the editing of the article.

## Supporting information

Additional supporting information may be found in the online version of this article: **Figure S1.** Histograms of individual-level residuals among boys. **Figure S2.** Histograms of individual-level residuals among girls. **Table S1.** Summary of how covariables might confound or mediate associations of maternal education with birth length and childhood height growth. **Table S2.** Association between confounders/mediators and outcomes among boys. **Table S3.** Association between confounders/mediators and outcomes among girls. **Table S4.** Mean predicted heights at birth and ages 2 and 4 years by category of maternal education.
