# Supplementary material for: Maternal education inequalities in height growth rates in early childhood: 2004 Pelotas birth cohort study
Source: Paediatr Perinat Epidemiol. 2012 Jan 13;26(3):236–49. doi: 10.1111/j.1365-3016.2011.01251.x (PMC3491696; doi:10.1111/j.1365-3016.2011.01251.x)

**Supporting information**

**Table S**1: Summary of how covariables might confound or mediate associations of maternal education with birth length and childhood height growth.

| Covariable | Expected (from literature) associations with exposure and outcome | Considered to be confounder (C), mediator (M), or possibly both (CM) |
| --- | --- | --- |
| Family income | Lower family income associated with lower maternal education but temporal nature unclear (i.e. lower educated mothers may be less likely to have income jobs or marry men with high income jobs OR women from lower background income families may have fewer educational opportunities).  Lower family income associated with shorter birth length and childhood stature | CM |
| Marital Status | Being unmarried is likely to act as an additional measure of SEP.  Women with lower education are less likely to be married. Unmarried (as a marker of lower SEP) may be associated with shorter birth length and childhood stature. | CM |
| Maternal age at birth | Women with lower education more likely to have children at a younger age.  Particularly young women (e.g. still in adolescence) who are still growing and developing themselves, more likely to have shorter infants at birth | M |
| Maternal parity | Women with lower education more likely to have greater parity.  Infant size at birth tends to increase with increasing parity. | M |
| Maternal skin colour | Skin colour would be an indicator of ethnicity.  Educational possibilities may vary by ethnicity.  Growth tends to differ by ethnicity (via genetic or lifestyle mechanisms) | C |
| Maternal height | Lower education attainment tends to be related to shorter stature. Therefore mothers with lower educational attainment (who may be from poorer backgrounds) would likely be shorter and their stature may limit intrauterine growth resulting in shorter birth length. This would therefore suggest maternal height was an environmental mediator.  Maternal height would also be linked to childhood length and growth via genetic variants as height is highly heritable. If there were assortative mating by educational attainment (or by other SEP indicators related to education) then maternal height could be a proxy for genetic confounding in the association of maternal education with offspring length/height growth. | CM |
| Maternal smoking in pregnancy | Women of lower education are more likely to smoke in pregnancy.  Smoking in pregnancy is associated with lower birth length and women who smoke in pregnancy are more likely to smoke postnatally, which is associated with childhood stunting (via passive smoking) | M |
| Gestational age | Preterm birth is more common in women from lower socioeconomic groups, including with lower education.  Earlier gestation is associated with shorter birth length. | M |
| Breast feeding | In developed countries lower maternal education is associated with reduced duration of breast feeding (though in some low and middle income countries the association can be in the opposite direction).  Breast feeding is associated with greater infant/childhood growth | M |

SEP= socioeconomic position

**Table S2 : Association b**etween confounders /mediators and outcomes among boys.

| Variables | Birth length (cm) | Growth 0-3 months (cm/month) | Growth 3-12 months (cm/month) | Growth 12-32 months (cm/month) | Growth 32-max months (cm/month) |
| --- | --- | --- | --- | --- | --- |
| Family income (MW)  ≤1.0  1.1 – 3.0  3.1 – 6.0  6.1 – 10.0  >10.0 | *p=0.077*  48.37 (0.13)  48.54 (0.13)  48.57 (0.13)  48.72 (0.12)  48.84 (0.12) | *p=0.001*  4.00 (0.04)  3.93 (0.03)  4.04 (0.03)  4.10 (0.03)  4.13 (0.03) | *p=0.061*  1.61 (0.01)  1.62 (0.01)  1.60 (0.01)  1.61 (0.01)  1.65 (0.01) | *p<0.001*  0.98 (0.01)  0.98 (0.01)  1.01 (0.01)  1.04 (0.01)  1.05 (0.01) | *p=0.942*  0.56 (0.01)  0.56 (0.01)  0.55 (0.01)  0.56 (0.01)  0.56 (0.01) |
| Marital status  Lived with partner  Single mother | *p=0.551*  48.60 (0.06)  48.69 (0.14) | *p=0.006*  4.06 (0.02)  3.95 (0.04) | *p=0.783*  1.62 (0.01)  1.62 (0.02) | *p=0.145*  1.01 (0.01)  1.00 (0.01) | *p=0.612*  0.56 (0.01)  0.56 (0.01) |
| Maternal skin colour  White  Black  Other | *p<0.001*  48.76 (0.06)  48.10 (0.13)  48.50 (0.22) | *p=0.327*  4.06 (0.02)  4.00 (0.04)  4.02 (0.06) | *p=0.237*  1.62 (0.01)  1.60 (0.01)  1.63 (0.02) | *p<0.001*  1.02 (0.01)  1.00 (0.01)  0.96 (0.02) | *p=0.001*  0.55 (0.01)  0.58 (0.01)  0.57 (0.01) |
| Maternal age (years)  ≤19  20-34  ≥35 | *p=0.047*  48.36 (0.13)  48.71 (0.07)  48.51 (0.16) | *p=0.256*  3.99 (0.04)  4.06 (0.02)  4.04 (0.04) | *p=0.974*  1.62 (0.01)  1.62 (0.01)  1.62 (0.02) | *p=0.003*  0.98 (0.01)  1.02 (0.01)  1.01 (0.01) | *p=0.786*  0.56 (0.01)  0.56 (0.01)  0.56 (0.01) |
| Maternal height (m)  <1.50  ≥1.50 | *p<0.001*  47.76 (0.21)  48.68 (0.06) | *p<0.001*  3.86 (0.06)  4.06 (0.02) | *p=0.027*  1.57 (0.02)  1.62 (0.01) | *p<0.001*  0.96 (0.02)  1.02 (0.01) | *p<0.001*  0.52 (0.01)  0.56 (0.01) |
| Parity  0  1  ≥2 | *p<0.001*  48.37 (0.09)  48.89 (0.11)  48.73 (0.10) | *p<0.001*  4.13 (0.02)  4.04 (0.03)  3.92 (0.03) | *p<0.001*  1.65 (0.01)  1.58 (0.01)  1.61 (0.01) | *p<0.001*  1.02 (0.01)  1.03 (0.01)  0.98 (0.01) | *p=0.265*  0.56 (0.01)  0.56 (0.01)  0.55 (0.01) |
| Maternal smoking during pregnancy  No  Yes | *p=0.001*  48.72 (0.07)  48.31 (0.11) | *p=0.012*  4.01 (0.02)  3.98 (0.03) | *p=0.209*  1.62 (0.01)  1.61 (0.01) | *p=0.001*  1.02 (0.01)  0.99 (0.01) | p=0.896  0.56 (0.01)  0.56 (0.01) |
| Gestational age (weeks)  <37  ≥37 | *p<0.001*  45.62 (0.14)  49.05 (0.05) | *p=0.128*  4.10 (0.04)  4.04 (0.02) | *p<0.001*  1.79 (0.02)  1.59 (0.01) | *p=0.669*  1.02 (0.01)  1.01 (0.01) | *p=0.266*  0.57 (0.01)  0.56 (0.01) |
| Duration of breastfeeding (months)  <3  ≥3 | *p<0.001*  48.29 (0.11)  48.73 (0.07) | *p=0.003*  3.97 (0.03)  4.07 (0.02) | *p<0.001*  1.69 (0.01)  1.59 (0.01) | *p=0.265*  1.00 (0.01)  1.01 (0.01) | *p=0.565*  0.55 (0.01)  0.56 (0.01) |

**Table S3 : Association between confounders /mediators and outcomes among girls.**

| Variables | Birth length (cm) | Growth 0-3 months (cm/month) | Growth 3-12 months (cm/month) | Growth 12-32 months (cm/month) | Growth 32-max months (cm/month) |
| --- | --- | --- | --- | --- | --- |
| Family income (MW)  ≤1.0  1.1 – 3.0  3.1 – 6.0  6.1 – 10.0  >10.0 | *p=0.001*  47.43 (0.13)  47.65 (0.13)  48.03 (0.13)  48.08 (0.12)  48.02 (0.13) | *p=0.005*  3.80 (0.03)  3.74 (0.03)  3.85 (0.03)  3.86 (0.03)  3.91 (0.03) | *p=0.001*  1.57 (0.01)  1.57 (0.01)  1.56 (0.01)  1.62 (0.01)  1.62 (0.01) | *p=0.001*   - 1. (0.01)   2. (0.01)   1.05 (0.01)  1.05 (0.01)  1.07 (0.01) | *p=0.606*  0.46 (0.01)  0.47 (0.01)  0.47 (0.01)  0.47 (0.01)  0.48 (0.01) |
| Marital status  Lived with partner  Single mother | *p=0.281*  47.87 (0.06)  47.70 (0.14) | *p=0.305*  3.82 (0.02)  3.86 (0.04) | *p=0.596*  1.59 (0.01)  1.58 (0.02) | *p=0.036*  1.06 (0.01)  1.02 (0.01) | *p=0.573*  0.47 (0.01)  0.48 (0.01) |
| Maternal skin colour  White  Black  Other | *p=0.548*  47.86 (0.07)  47.74 (0.13)  47.98 (0.21) | *p=0.016*  3.86 (0.02)  3.77 (0.03)  3.75 (0.05) | *p=0.616*  1.59 (0.01)  1.58 (0.01)  1.59 (0.02) | *p<0.001*  1.05 (0.01)  1.01 (0.01)  1.04 (0.01) | *p<0.001*  0.46 (0.01)  0.50 (0.01)  0.49 (0.01) |
| Maternal age (years)  ≤19  20-34  ≥35 | *p=0.156*  47.65 (0.13)  47.87 (0.07)  48.02 (0.16) | *p=0.919*  3.82 (0.03)  3.83 (0.02)  3.83 (0.04) | *p=0.634*  1.58 (0.01)  1.59 (0.01)  1.59 (0.02) | *p=0.047*  1.02 (0.01)  1.04 (0.01)  1.06 (0.01) | *p=0.409*  0.48 (0.01)  0.47 (0.01)  0.48 (0.01) |
| Maternal height (m)  <1.50  ≥1.50 | *p<0.001*  46.37 (0.20)  48.00 (0.06) | *p=0.011*  3.70 (0.05)  3.84 (0.02) | *p=0.015*  1.54 (0.02)  1.59 (0.01) | *p=0.003*  1.00 (0.01)  1.04 (0.01) | *p=0.080*  0.45 (0.01)  0.47 (0.01) |
| Parity  0  1  ≥2 | *p=0.073*  47.68 (0.09)  47.92 (0.11)  47.97 (0.10) | *p<0.001*  3.89 (0.02)  3.89 (0.03)  3.71 (0.02) | *p<0.001*  1.62 (0.01)  1.59 (0.01)  1.56 (0.01) | *p=0.001*  1.06 (0.01)  1.04 (0.01)  1.02 (0.01) | *p=0.080*  0.47 (0.01)  0.48 (0.01)  0.46 (0.01) |
| Maternal smoking during pregnancy  No  Yes | *p<0.001*  48.01 (0.07)  47.38 (0.11) | *p=0.001*  3.86 (0.02)  3.75 (0.03) | *p=0.956*  1.59 (0.01)  1.59 (0.01) | *p=0.001*  1.05 (0.01)  1.01 (0.01) | *p=0.554*  0.47 (0.01)  0.47 (0.01) |
| Gestational age (weeks)  <37  ≥37 | *p<0.001*  45.13 (0.14)  48.29 (0.06) | *p=0.004*  3.93 (0.04)  3.81 (0.02) | *p<0.001*  1.69 (0.02)  1.57 (0.01) | *p=0.762*  1.04 (0.01)  1.04 (0.01) | *p=0.944*  0.47 (0.01)  0.47 (0.01) |
| Duration of breastfeeding (months)  <3  ≥3 | *p=0.001*  47.49 (0.11)  47.97 (0.07) | *p=0.544*  3.82 (0.03)  3.84 (0.02) | *p<0.001*  1.65 (0.01)  1.57 (0.01) | *p=0.111*  1.03 (0.01)  1.04 (0.01) | *p=0.247*  0.48 (0.01)  0.47 (0.01) |

**Table S4: Mean predicted heights at birth and ages two and four years by category of maternal education**

|  | Mean (SE) predicted height by level of Maternal education | | |
| --- | --- | --- | --- |
|  | 0-4 years | 5-8 years | 9+ years |
| Boys |  |  |  |
| Birth | 48.21 (0.15) | 48.54 (0.08) | 48.80 (0.08) |
| Age 2 years | 85.74 (0.22) | 86.99 (0.13) | 88.36 (0.12) |
| Age 4 years | 101.06 (0.28) | 102.47 (0.16) | 104.20 (0.15) |
|  |  |  |  |
| Girls |  |  |  |
| Birth | 47.54 (0.15) | 47.81 (0.09) | 47.97 (0.09) |
| Age 2 years | 84.49 (0.22) | 85.69 (0.13) | 86.99 (0.12) |
| Age 4 years | 100.02 (0.27) | 101.43 (0.16) | 103.03 (0.15) |

SE: standard error

**Figure S1 – Histograms of individual level residuals among boys**


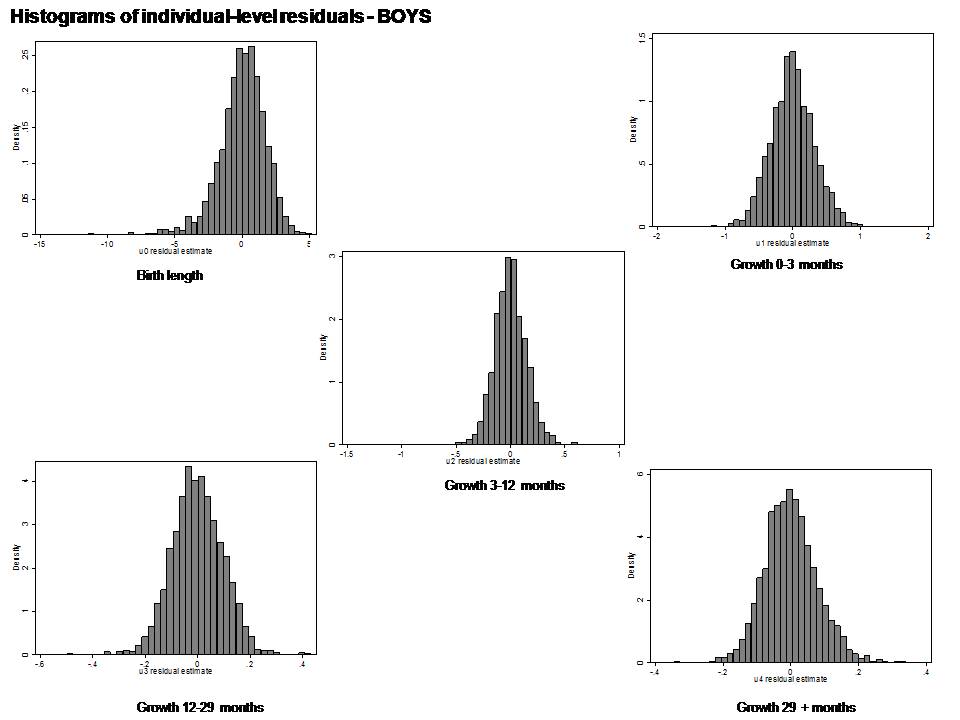


**Figure S2 – Histograms of individual level residuals among girls**


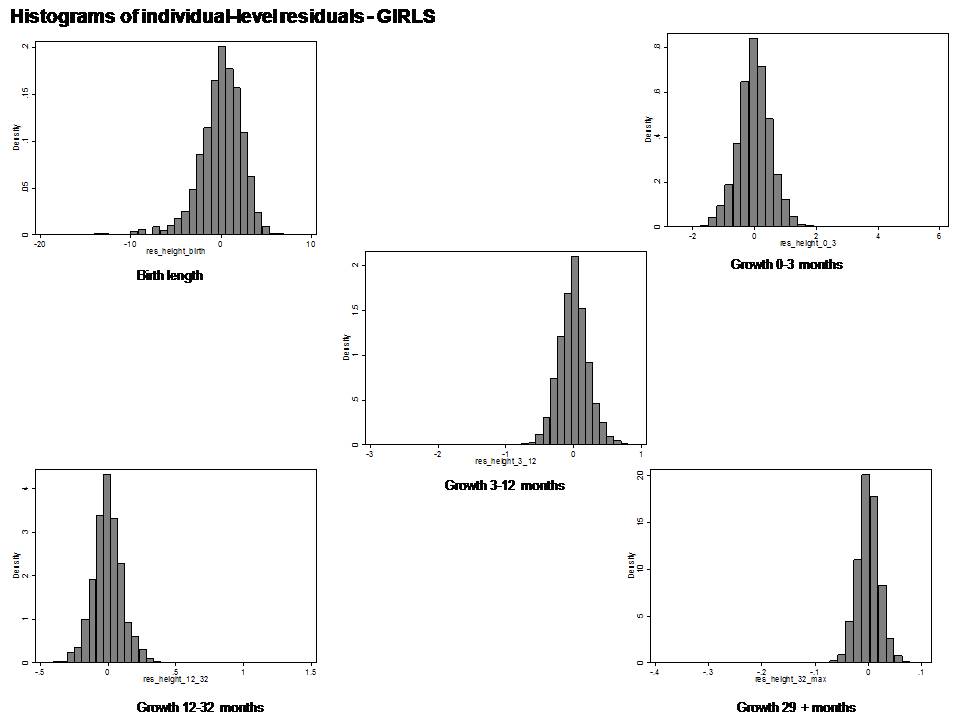

Supplement: Supplementary file 1 — Additional supporting information may be found in the online version of this article: Figure S1. Histograms of individual-level residuals among boys. Figure S2. Histograms of individual-level residuals among girls. Table S1. Summary of how covariables might confound or mediate associations of maternal education with birth length and childhood height growth. Table S2. Association between confounders/mediators and outcomes among boys. Table S3. Association between confounders/mediators and outcomes among girls. Table S4. Mean predicted heights at birth and ages 2 and 4 years by category of maternal education. [file ppe0026-0236-SD1.doc]
